# Does Parental Presence Influence Child Performance on an Emotional Go/No‐Go Task at Age 9.5? Exploring the Role of Puberty and Early Environmental Quality

**DOI:** 10.1111/desc.70206

**Published:** 2026-04-26

**Authors:** Saara Nolvi, Laura Perasto, Pauliina Juntunen, Tuomo‐Artturi Autere, Venla Huovinen, Aino Luotola, Hilyatushalihah Kholis Audah, Max Karukivi, Anna‐Katariina Aatsinki, Hasse Karlsson, Linnea Karlsson, Minna Lukkarinen, Nim Tottenham, Riikka Korja

**Affiliations:** ^1^ Department of Psychology and Speech‐Language Pathology University of Turku Turku Finland; ^2^ The Centre of Excellence for Learning Dynamics and Intervention Research (InterLearn) University of Turku, Turku, and University of Jyväskylä, Jyväskylä Finland; ^3^ FinnBrain Birth Cohort Study, Turku Brain and Mind Center, Department of Clinical Medicine University of Turku and Turku University Hospital Turku Finland; ^4^ Centre for Population Health Research University of Turku and Turku University Hospital Turku Finland; ^5^ Department of Adolescent Psychiatry University of Turku and Turku University Hospital Turku Finland; ^6^ Department of Psychiatry University of Turku and Turku University Hospital Turku Finland; ^7^ Department of Public Health University of Turku and Turku University Hospital Turku Finland; ^8^ Department of Child Psychiatry University of Turku and Turku University Hospital Turku Finland; ^9^ The Department of Pediatrics and Adolescent Medicine University of Turku and Turku University Hospital Turku Finland; ^10^ Department of Psychology Columbia University New York New York USA

**Keywords:** buffering, caregiving, emotion regulation, executive function, parental presence, puberty

## Abstract

Previous research suggests that children may perform better and regulate emotions more effectively in the presence of a parent than an unfamiliar stranger, a phenomenon known as parental buffering. This reliance may vary with pubertal development and early psychosocial environments. However, existing studies often rely on small, mixed‐age samples and provide limited insight into how normative variation in caregiving quality and parental mental health influences children's transition from parental dependence to independent regulation before puberty. In this pre‐registered study, we examined children's performance on an emotional go/no‐go task under parent‐present versus stranger‐present conditions in a large sample of 9.5‐year‐old children (*N* = 501) from the FinnBrain Birth Cohort Study. In smaller subsamples, we tested whether pubertal stage affected parental effects on performance and whether early maternal caregiving quality and long‐term parental distress moderated these effects. We did not find consistent evidence for parental buffering of 9.5‐year‐olds’ performance in either pre‐pubertal or pubertal children. However, sensitivity analyses including all trials, with effect sizes resembling those of the main models, suggested that children made fewer errors when a parent (vs. a stranger) was present, consistent with our pre‐registered hypotheses. Evidence for moderation by early caregiving quality or parental mental health was minimal. These findings highlight the need for longitudinal, age‐specific research on children's reliance on parental presence for emotion regulation and suggest that typical variation in caregiving quality and parental mental health may not substantially influence parental buffering effects in middle childhood.

## Summary

- We found limited evidence for buffering effects on child performance during an emotional go/no‐go task in 9.5‐year‐old children, regardless of pubertal status.
- Sensitivity analyses suggested that children made fewer errors in the presence of their parents.
- Long‐term maternal caregiving quality and parental distress did not moderate parental buffering effects.
- The results underscore the need for longitudinal research to explore age‐specific changes and mechanisms in children's reliance on parents for emotion regulation.

## 1 Introduction

Previous studies have shown that mere parental presence can buffer children's physiological stress responses, such as cortisol secretion, even in stressful situations (Doom et al. 2015; Hostinar et al. 2015). Parental regulation of physiological needs also serves as a foundation for attachment formation, which is pivotal for offspring survival. This function is particularly important in humans, given the extended period of dependence on caregivers in comparison to other species (Tottenham 2020). Consistent with findings on corticosteroid secretion, recent research indicates that parental presence also modulates neural circuits involved in emotion regulation and fear conditions in children (Abramson et al. 2024; Day et al. 2020). These findings suggest that broad neurobiological systems related to self‐regulation are influenced by parental cues, extending beyond the well‐documented effects of specific parenting behaviors such as sensitivity or harshness (Farber et al. 2020; Morawska et al. 2019).

Interestingly, a series of studies also suggests that parental presence influences not only biological systems but also observable child behaviors (Gee et al. 2014; Tottenham et al. 2019). Building on earlier animal research (Moriceau and Sullivan 2006), Gee et al. (2014) demonstrated that child behavioral regulation during the emotional go/no‐go task improves in the presence of the parent compared to the presence of an unfamiliar stranger. Furthermore, prior research indicates that these behavioral differences are no longer observed in adolescents (Gee et al. 2014; Hostinar et al. 2015; Moriceau and Sullivan 2006), suggesting that puberty may modulate this effect (Doom et al. 2015). It has therefore been proposed that parental presence has a specific modulatory effect on child behavior up until approximately Ages 9–10, before the onset of puberty in most children. However, existing studies have typically been conducted in small samples within experimental neuroscience settings, and findings have yet to be robustly replicated in larger cohorts. It also remains unclear whether the buffering effect of parental presence is consistent across all prepubertal children, or whether more nuanced age‐related variations exist. A deeper understanding of the conditions under which parental presence influences child behavioral regulation is essential for clarifying the role of parenting across childhood and adolescence—particularly during the transition to adolescence, a period marked by increased vulnerability to emotional dysregulation and mental health difficulties (Blakemore 2019).

Additionally, there is substantial evidence suggesting that poorer early environmental quality or higher adversity may contribute to an earlier onset of puberty (Ellis et al. 1999; Hamlat et al. 2023; Pham et al. 2022; Thijssen et al. 2020), as proposed by theories of accelerated development and aging (Belsky et al. 1991). This association is further supported by findings that both early pubertal timing and adverse environmental conditions are linked to poorer health and well‐being across the lifespan (Dehestani et al. 2023; Dimler and Natsuaki 2015; Hughes et al. 2017; Ullsperger and Nikolas 2017). In relation to potential parental buffering effects, research has shown that early adversity may narrow the developmental “window” during which parental presence effectively regulates neural and hormonal responses. Thus, children exposed to such environments may become less reliant on parental cues for physiological regulation (Gee et al. 2013; Perry et al. 2021; Thijssen et al. 2017) or they may exhibit atypical behavioral responses in the presence of their parents (Wismer Fries et al. 2008). However, these studies have primarily focused on cortisol reactivity to stressors (Perry et al. 2021; Wismer Fries et al. 2008), or neural activation patterns relevant to emotion regulation, such as medial prefrontal cortex–amygdala connectivity (Gee et al. 2013; Thijssen et al. 2017). To date, we are not aware of studies that have examined these effects at the behavioral level, such as performance on self‐regulation tasks. Moreover, most prior research has investigated extreme forms of early adversity, such as institutional care followed by adoption (Gee et al. 2013; Perry et al. 2021; Wismer Fries et al. 2008), with only one study suggesting that normative variation in parenting (e.g., sensitivity vs. insensitivity) also modulates brain connectivity relevant for emotion regulation during the transition to adolescence (Thijssen et al. 2017). Consequently, little is known about how more typical variations in the early family environments that generally represent low socioeconomic and psychopathology risk influence the role of parental presence in child emotion regulation at the behavioral level, particularly in the context of long‐term familial conditions. Additionally, existing studies have generally relied on small samples, limiting the statistical power to detect reliable moderation effects.

In this study, we aim to address these gaps by looking at two longitudinal indices of early environmental quality: First, caregiving quality, assessed through observations of maternal emotional availability (EA; Biringen 2008) across three time points in early childhood, and second, the level of parental depressive and anxiety symptoms measured repeatedly throughout childhood. EA refers to a caregiver's ability to respond sensitively and attune to a child's needs and goals while accepting a wide range of emotional expressions by the child. It has been associated with positive developmental outcomes in early childhood (Biringen et al. 2014), providing a reliable indicator of caregiving quality across early development. Furthermore, EA reportedly provides a reliable measure of at‐risk caregiving behaviors, such as high hostility, intrusiveness, and insensitivity, which are indicative of poor caregiving quality and poor early environmental quality. In parallel, chronic parental symptoms of depression and anxiety have been strongly linked to child emotion dysregulation and adverse mental health outcomes over time (Korja et al. 2024; Lawrence et al. 2019; Rees et al. 2019; Tu et al. 2024). Early childhood is a particularly sensitive period for the development of self‐regulatory skills, as neural systems are highly responsive to environmental input. Therefore, assessing environmental factors during this stage—particularly through repeated measures—provides valuable insights into how the early family environment shapes the effectiveness of parental presence in supporting emotion regulation, particularly during the transition to adolescence.

In the present study, following our pre‐registration (https://doi.org/10.17605/OSF.IO/CDNH7), we used a general population sample of children with a mean age of 9.5 years, drawn from the longitudinal FinnBrain Birth Cohort Study, to investigate (1) whether parental presence affects child task performance on an emotional go/no‐go regulation task compared to the presence of an unfamiliar experimenter at this specific age, and (2) whether early life environmental quality, as characterized by lower maternal EA and chronic maternal depressive and anxiety symptoms, moderates the effect of parental versus stranger presence on task performance. As outlined in the pre‐registration, we first investigated the differences between the parent and stranger conditions without covariates and subsequently while accounting for child age and biological sex. Models examining modulation by environmental quality were conducted in a similar manner, but controlling also for maternal demographic characteristics. In contrast to the pre‐registered plan, because information on pubertal stage was available only for a substantially smaller subsample, we conducted sensitivity analyses controlling for pubertal stage and excluding children who had entered puberty. Additionally, although not specified in the pre‐registration, we performed sensitivity analyses stratified by child biological sex. Finally, because the data‐processing criteria applied (e.g., exclusion of trials with low accuracy) were not available at the time of pre‐registration, we conducted an additional sensitivity analysis including all trials regardless of accuracy. As an exploratory, non‐pre‐registered analysis—though noted in the pre‐registration plan—we investigated whether paternal psychological distress, measured via symptoms of depression and anxiety, moderated child performance.

We hypothesized that children would perform differently when a parent was present compared to when an unfamiliar experimenter was present. Furthermore, we expected that lower environmental quality would moderate this effect, such that children exposed to higher maternal (and paternal) depressive and anxiety symptoms, as well as lower observed maternal EA, would show no significant difference between the parent and stranger conditions.

## 2 Methods

### 2.1 Participants and Procedures

The sample for this study is a part of a longitudinal FinnBrain Birth Cohort Study (Karlsson et al. 2018). The families (*N* = 3,808) were originally recruited during the ultrasound assessment at 12 weeks’ gestation through a personal contact by a research nurse. The children have been followed up from neonatal age, with some families invited to developmental visits throughout childhood, including a visit at the age of 9.5 years. Families who had participated in earlier developmental assessments or other visits at the 9 years of age were invited to a developmental visit at the child's age 9.5 years (±3 months) via personal contact from research personnel. Later, the sample was randomly enriched with families from the main cohort who had not attended previous research visits to ensure maximal sample size. The exclusion criterion for the visit was the child having been diagnosed with severe developmental disability (e.g., unable to communicate by speech, not recognizing letters).

The subsample for this study included children who participated in the emotional go/no‐go task during the 9.5‐year visit (*N* = 501). For the analysis relating to the question examining moderation by early life environmental quality, only families with available longitudinal data on maternal EA (*N* = 319) and maternal/paternal depressive and anxiety symptoms were included (*N* = 489 for mothers and *N* = 297 for fathers). Additionally, analyses examining the role of puberty in modulating the associations were conducted using a subsample due to missing puberty assessments (e.g., too long an interval between the visits caused by COVID‐19) in ca. half of the sample (*N* = 268).

The demographic and descriptive characteristics of the sample are presented in Table 1. The participants in the current study were comparable to the baseline cohort in terms of child biological sex assigned at birth, monthly income, financial satisfaction reported during pregnancy, and depressive and anxiety symptoms during mid‐ and late pregnancy. However, compared to the baseline cohort, the mothers and fathers in the current sample were slightly older and more highly educated (*p*s < 0.001), and the mothers reported lower levels of depressive and anxiety symptoms at the beginning of pregnancy (*p*s < 0.05). The subsample with available information on pubertal stage differed slightly from the full study sample in age (*p* = 0.029), with those children being slightly younger (*M* [SD] = 9.52 [0.10]) than the children in the full sample (*M* [SD] = 9.54 [0.10]). There were no significant differences between samples in terms of child sex, parental age, education, financial satisfaction, maternal caregiving, or parental depressive and anxiety symptoms across the follow‐up period.

**TABLE 1:** The sociodemographic characteristics of the sample.

	*N*	Range	*M* (SD)	No. (%)
Child characteristics				
Child biological sex assigned at birth, boy	501			258 (51.5)
Child age at visit (years)	501	9.33–10.00	9.53 (0.10)	
Pubertal stage (from those available)	268			
Boys, ≥G2P2^a^				5 (3.4)
Girls, ≥ M2P2^a^				23 (19.0)
Parental characteristics				
Parent role, mother				445 (88.8)
Maternal age at delivery		19.0–42.0	31.26 (4.58)	
Paternal age at delivery		18.0–57.0	32.86 (5.34)	
Maternal financial satisfaction^b^				
5 years		1–15	9.84 (3.33)	
9 years		0–10	6.00 (2.45)	
Paternal financial satisfaction^b^				
5 years		1–15	9.88 (3.38)	
9 years		0–10	6.40 (2.22)	
Maternal education, highest by 5 y				
<High school/Vocational education				122 (25.1)
Vocational tertiary/Applied university				143 (29.3)
University degree				222 (45.6)
Paternal education, highest by 5 y				
<High school/Vocational education				112 (35.0)
Vocational tertiary/Applied university				102 (31.9)
University degree				106 (33.1)
In subsamples with environmental modulators				
Maternal EA (intercept scores^c^)	319	−1.28–0.81	0.02 (0.41)	
Maternal depressive symptoms, avg.^d^	488	0–21	4.91 (3.51)	
Paternal depressive symptoms, avg.^d^	298	0–23	4.15 (3.66)	
Maternal anxiety symptoms, avg.^d^	489	0–19	3.27 (3.52)	
Paternal anxiety symptoms, avg.^d^	295	0–15	2.51 (2.84)	

^a^ Based on Tanner stages scale.

^b^ Standardized average of 5 and 9 yrs.

^c^ Direct scores of EA subscales at each time point are provided in the Supporting Information.

^d^ Depressive symptoms were measured using the EPDS, and anxiety symptoms were measures using the SCL‐90 anxiety subscale. The scores are aaw average scross across 6, 12 (EPDS only), 24 months, and 4, 5, and 9 years postpartum from those parents who have provided at least one report of symptoms.

### 2.2 Power Analysis

Based on previous literature (Gee et al. 2014), the estimated difference between the means in false alarm rates between parent/stranger conditions was 0.2 (SD = 0.15). A power analysis was carried out using these values and assuming a paired samples *t*‐test, showing that a sample size of 10 would provide 96% statistical power. Given that the sample size for this study (across all subsamples) was *N* > 100, the statistical power is deemed sufficient. Power calculations were performed using G*Power (Faul et al. 2007).

### 2.3 General Procedures of the Visit

The visit was altogether 3 h in length and included a set of neurocognitive tasks and questionnaires for the child as well as interaction tasks for the child and the caregiver, with two breaks during the visit. The visits were administered by clinical psychologists or advanced psychology students receiving supervision. Both parents and the child themselves gave their consent for participation at 9.5 years of age. The Ethics Committee of the Hospital District of Southwest Finland (the Ethics Committee for the Wellbeing Services County of Southwest Finland)  has approved the FinnBrain Study, the 9.5‐year visit, and previous visits whose data are used in the present study. This study has been performed in accordance with the ethical standards laid down in the 1964 Declaration of Helsinki and its later amendments.

### 2.4 Measures

#### 2.4.1 The Emotional Go/No‐Go Task

A version of the emotional go/no‐go task (Gee et al. 2014; Tottenham et al. 2011) was implemented as the second last section of the visit. Children viewed facial expressions on a computer screen and were instructed to press a button when a target facial expression (go) appeared, inhibiting this response when a distractor facial expression (no‐go) was shown. In the present study, the stimuli consisted of black‐and‐white images of six female and four male faces from the Ekman Pictures of Facial Affect database (Ekman and Friesen 1976), each displaying happy, fearful, angry, and neutral expressions. The visual angle was approximately 12°, and the images were presented in the center of the screen.

**FIGURE 1:**
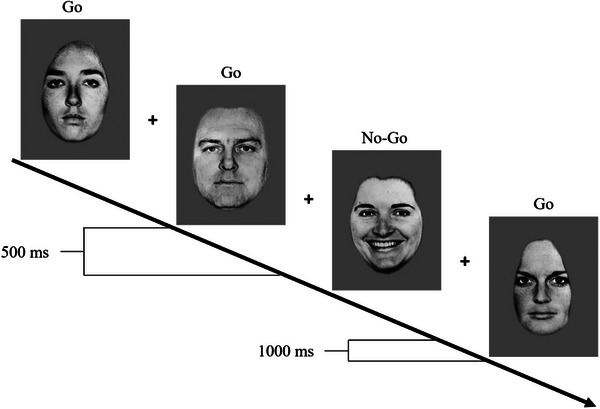
Illustration of the go–no‐go task, with neutral faces as the go stimulus and happy faces as the no‐go stimulus.

A practice trial was conducted to ensure participants understood the task instructions. The task comprised of the following six blocks: angry face (go)–neutral face (no‐go); fearful face (go)–neutral face (no‐go), happy face (go)–neutral face (no‐go); neutral face (go)–angry face (no‐go); neutral face (go)–fearful face (no‐go); and neutral face (go)–happy face (no‐go), with neutral and emotion alternating as the go stimulus. Participants were instructed to press the response button quickly for the go face, but they were not told which expression would serve as the no‐go stimulus. Blocks were counterbalanced across participants. The go facial expression appeared 70% of the time to establish a prepotent tendency to respond. Stimuli were presented in a random order for all participants. Each face stimulus was presented for 500 ms, followed by a 1000‐ms fixation period to allow adequate time for response, as illustrated in Figure 1. Stimulus presentation and response collection were performed using E‐Prime software on a Dell computer.

The task was administered twice: once in the presence of the participant's parent (mother in 89% of experiments) and once without the parent, in the presence of a stranger (a research assistant who was present for both sessions to administer the task). During each session, the research assistant, or both the parent and the research assistant, sat next to the participant while they completed the task. Participants were instructed to focus on the task in both conditions. Prior to beginning the task, parents were informed—while in a separate room without the child present—that the purpose of the session was to study the effects of parental presence. They were instructed not to speak or interact with the participant during the task but instead to complete a questionnaire on a tablet. The order of administration (parent‐first or stranger‐first condition) was counterbalanced.

We used reaction time, false alarm rates, and hit accuracy rates as outcome variables, as outlined in the pre‐registration. For each participant, we calculated hit accuracy as the difference between the number of hits in response to go faces and the number of false alarms (i.e., responding to a no‐go face). We calculated the mean reaction time for hits in response to neutral faces in the context of happy no‐go faces.

#### 2.4.2 Early Life Environmental Quality: Maternal Caregiving Quality in Early Childhood

Mother–child interactions were recorded when the child was 8 months, 2.5 years, and 5 years old. Procedures varied slightly across time points, including a 20‐min free‐play session at 8 months; a 15‐min free‐play and a 5‐min snack period at 30 months; and a 15‐minfree‐play, 5‐min snack period, and 5‐min structured task at 5 years, of which only the free play was analyzed at 5 years. The play materials provided at each time point were age‐appropriate. Interactions were coded using the EA Scales (Biringen 2008), which assess maternal EA across the following four dimensions: sensitivity, structuring, nonintrusiveness, and nonhostility. Each dimension is rated on a 7‐point scale, with higher scores indicating greater EA. Scores between 5.5 and 7 reflect an emotionally available and healthy mother–child relationship, whereas scores between 1 and 5 indicate varying degrees of problematic EA (Biringen 2008). Coding was performed by trained raters who were blind to other study data. Inter‐rater reliability was high, with intraclass correlation coefficients (ICCs) ranging from 0.80 to 0.91 for sensitivity, 0.72 to 0.91 for structuring, 0.81 to 0.90 for nonintrusiveness, and 0.70 to 0.85 for nonhostility. Drawing on prior findings from the current project that demonstrate longitudinal stability in maternal EA during early childhood (Juntunen et al. submitted), a second‐order intercept‐only Latent Growth Model (LGM) was constructed. This model included the four EA dimensions assessed across the three time points. The model demonstrated acceptable fit across multiple indices, namely root mean square error of approximation (RMSEA), comparative fit index (CFI), and square root of mean differences (SRMR): (*χ* ^2^(48) = 64.37, *p* = 0.057, RMSEA = 0.028, CFI = 0.991, and SRMR = 0.080). The resulting intercept factor from the LGM was used in subsequent analyses as a composite estimate of overall maternal EA.

#### 2.4.3 Early Life Environmental Quality: Maternal and Paternal Psychological Distress Throughout Childhood

Maternal symptoms of depression were assessed using the widely used and validated Edinburgh Postnatal Depression Scale (the EPDS; Cox et al. 1987). The EPDS comprises 10 items, with respondents rating the frequency of symptoms experienced over the past week on a 4‐point scale ranging from 0 to 3. Total scores range from 0 to 30, with higher scores indicating greater levels of depressive symptoms. Anxiety symptoms were assessed using the 10‐item anxiety subscale of the Symptom Checklist‐90 (Derogatis et al. 1973; Holi 2003), a well‐established measure commonly used in both clinical and research contexts. Each item is rated on a 6‐point scale from 0 to 5, yielding a total score ranging from 0 to 50, with higher scores reflecting more severe anxiety symptoms. Data were collected at seven time points: when the child was 3, 6, 12, and 24 months, and 4, 5, and 9 years of age. The EPDS and SCL‐90 demonstrated good internal consistency throughout the follow‐up (alphas ranging from 0.80 to 0.87 for the EPDS and from 0.80 to 0.88 for the SCL). As a deviation from the original plan, to retain the highest possible power, all the parents with reports from any time point were used, and missing values across follow‐up were multiple imputed. The symptom scores were standardized and aggregated to derive a composite measure of overall psychological distress experienced by the parent across the follow‐up period.

#### 2.4.4 Covariates

Child birth date used to calculate child age and child biological sex assigned at birth (later referred to as sex, 1 = boy, 2 = girl) were drawn from the hospital records of the Wellbeing Services County of the Southwest Finland  after the child was born. Additional background variables used in moderator analyses were collected via maternal and paternal self‐reports. Parental age was recorded at baseline (gestational week 12), while educational attainment was determined based on the highest level of education reported by either parent by the child's age of 5, supplemented with information collected during gestational week 14 (1 = low/secondary education; 2 = middle/applied university degree; 3 = high/university degree). Financial satisfaction was measured as the mean of parental satisfaction ratings collected during pregnancy and at the child's age of 5 years. Ratings were provided on a continuous scale from 0 to 10 during pregnancy and from 0 to 15 at Age 5. These scores were standardized and combined to create a single measure of overall financial satisfaction.

In a subsample, the stage of puberty was defined based on Tanner stages that were evaluated during a separate study visit when the children were approximately 9.5 years. Pubertal development was assessed by a trained research nurse or by parental‐ or self‐report, based on cartoon figures if the child denied assessment by a nurse. Girls exhibiting breast development at stage 2 (M2) and/or pubic hair at stage 2 (P2), and boys exhibiting genital development at stage 2 (G2) and/or pubic hair at stage 2 (P2)—with either measure rated ≥2—were classified as having entered puberty (coded as 1). Children having this assessment available prior to or less than 2 months after the developmental visit were included in the subsample. In turn, children who received a score of 1 on both relevant measures and had an assessment available within 2 months before or any time after the developmental visit were classified as not having entered puberty (coded as 0).

### 2.5 Analysis Strategy

The pre‐registered analysis plan was published prior to conducting any analyses and is available at 2014 (https://doi.org/10.17605/OSF.IO/CDNH7). Based on careful examination of the data and prior practices concerning reaction times, all trials with reaction times faster than 200 ms were excluded from the analysis. Consistent with prior practices, we also conducted all the analyses excluding the go‐trials in which the participant's accuracy was below 70%. However, because this criterion was not specified in the pre‐registration, we also conducted sensitivity analyses including all go trials. Benjamini and Hochberg FDR‐corrected *p* values (two‐tailed) smaller than 0.05 were considered statistically significant. Due to skewness in the distribution of model residuals, the bias‐corrected beta coefficients (estimates) and 95% bootstrap percentile confidence intervals (CIs) were reported (based on 1000 bootstrap samples). Analyses were performed in R (4.2.2, 2022) using the packages boot, lme4, and ggplot2 (the scripts are provided at https://osf.io/3v2d7/files/osfstorage).

#### 2.5.1 Modeling

As outlined in the pre‐registration, repeated‐measures general linear models were initially conducted to examine differences between the parental and stranger conditions in false alarm rates, hit rates, and reaction times. However, because a linear mixed‐modeling approach that tests condition differences while controlling for emotion and block type effects better accounts for the data structure, and because both analytic approaches yielded similar patterns of results, only the mixed models are presented in the main manuscript, with the original repeated‐measures GLMs reported in the Supporting Information.

The following formula was used for each outcome in the linear mixed‐effects models:

$$\begin{array}{ccc} & & \mathrm{outcome}∼\mathrm{intercept}+\mathrm{emotion}\times \mathrm{block}+\mathrm{condition}+(1|\mathrm{ID})\\ & & +\left(1|\mathrm{session}\mathrm{order}\right), \end{array}$$

where emotion (fear, angry, happy), block (emotion as go or no‐go), condition (parent or stranger present), and session order (first with stranger or with parent) were treated as categorical variables. Random effects for the child and session order were included to account for repeated measures from the same individuals. The interaction between emotion and block was included to account for differences in the child's outcomes, depending on whether a specific emotion was a go or no‐go signal.

Following the pre‐registration, the models were first run without covariates and subsequently with the inclusion of child age and biological sex assigned at birth, in order to retain maximal statistical power in the primary models. In contrast to the pre‐registered plan, analyses controlling for the pubertal stage and excluding the children who had entered puberty from the sample were treated as a sensitivity analysis because puberty information was available only for a substantially smaller subsample. Additional, non‐pre‐registered sensitivity analyses examined the effects stratified by sex. As indicated above, analyses were also run including all go trials regardless of accuracy, a criterion that was not specified in the pre‐registration. The gender of the parent present during the task did not affect the pattern of results, and therefore, those results are not reported separately.

For the second research question examining moderation by early life environmental quality, we used similar general linear models (see Supporting Information) and linear mixed effects models with two moderating variables: (1) maternal EA intercept or (2) maternal psychological distress as moderators. These models included the same covariates identified earlier (age and sex, with pubertal stage included in the smaller subsample). Not specified in the pre‐registration, these models were also controlled for maternal education and economic satisfaction (indicators of socioeconomic status [SES]) and maternal age that were considered potential confounders related to both child emotion regulation and parental caregiving/psychological distress. In our data, these covariates were linked to almost all child outcomes and maternal moderators (see Table S4 in the Supporting Information).

#### 2.5.2 Exploratory Analyses

Following the pre‐registered plan, an exploratory linear mixed effects (or repeated GLM) model was run using paternal psychological distress, measured as depressive and anxiety symptoms, in a smaller subsample of the participants with paternal symptom data. These models were controlled for father‐specific SES and age covariates, which were related to child outcome variables in a similar manner to the corresponding maternal variables (see Table S5 in the Supporting Information).

## 3 Results

### 3.1 Descriptive Statistics

The associations between the main variables of the study are presented in Table 2. There were modest, expected correlations between the false alarm rates, go accuracy, and reaction times across the dataset. Higher age was marginally associated with faster reaction times but was not related to other performance variables. Participants identified as girls at birth showed slower reaction times but made fewer mistakes and were slightly more accurate on go trials than participants identified as boys. The associations were nearly identical when analyses were restricted to participants with available pubertal stage information or when excluding the children who had entered puberty, and were consistent across both sessions (see Table S2 in the Supporting Information). In addition, several expected emotion‐specific associations were observed, further supporting the use of linear mixed models as the primary analytic approach (see Table S2).

**TABLE 2:** Spearman correlation coefficients between the main performance and background variables when including all the trials (*n* = 501).

	1	2	3	4
1. Reaction time	1			
2. False alarm rate	−0.331^***^	1		
3. Accuracy (hits)	−0.097^***^	0.104^***^	1	
4. Age of the child	−0.030^*^	−0.005	−0.011	1
5. Sex of the child^a^	0.196^***^	−0.183^***^	0.082^***^	−0.044^**^

^a^ Levels: 0 = boys, 1 = girls.

* *p* < 0.05.

** *p* < 0.01.

*** *p* < 0.001.

### 3.2 Differences in Child Performance Between Parent Versus Stranger Presence

**FIGURE 2:**
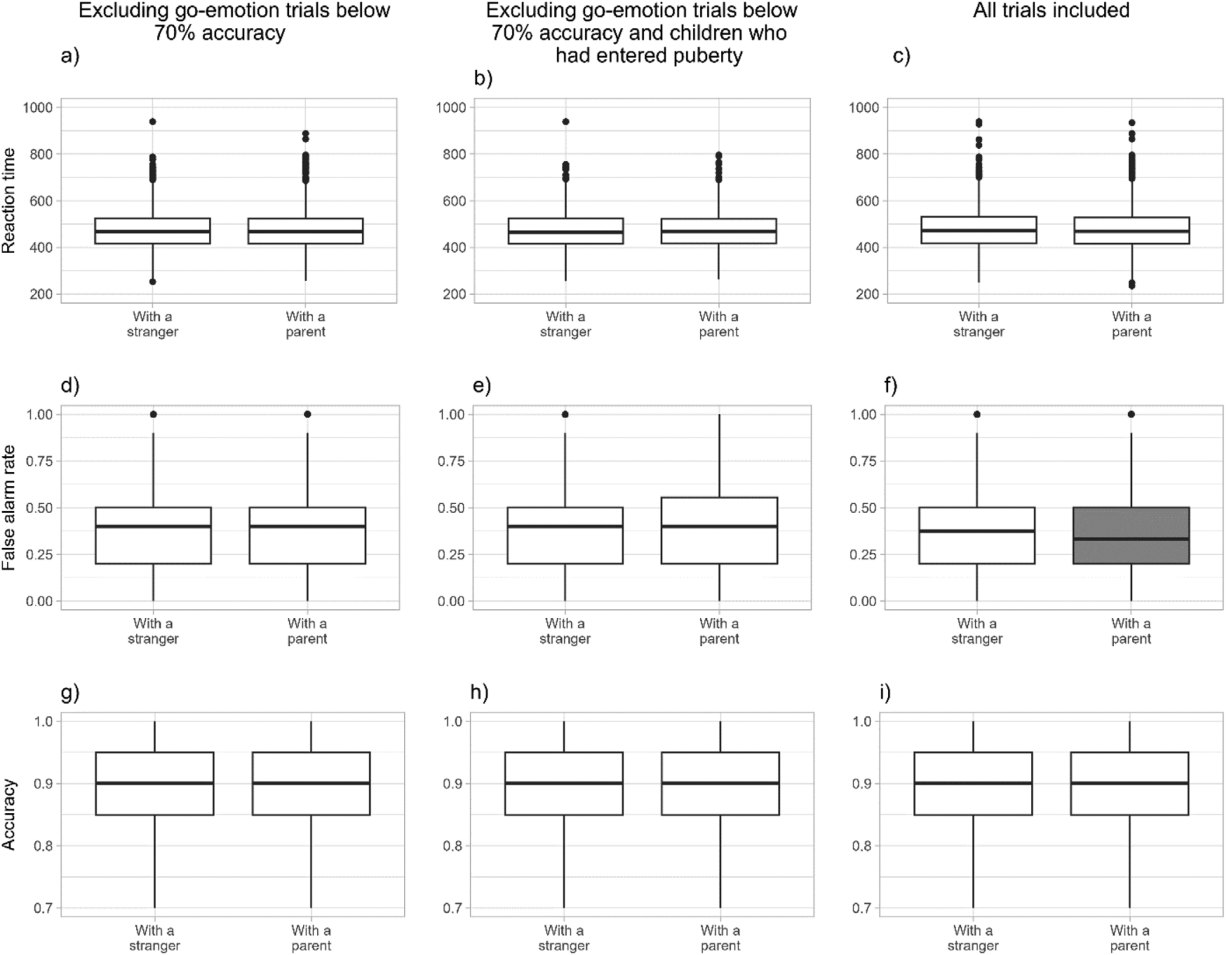
The difference between parent versus stranger conditions in child reaction times, false alarm rates and accuracy rates across stranger and parent conditions in the full dataset ((*N* = 501, see a, d, g), in the subsample where the children who have entered puberty were removed (*N* = 240, see b, e, h) and in the full sample but including all go trials (see c, f, i). The significant condition difference is highlighted in gray.).

In the full dataset, after controlling for emotion and block type effects, there were no significant differences in child reaction times, false alarm rates, or accuracy between parent versus stranger conditions. Similarly, in the model controlling for child age and sex, we observed no differences in child performance between parent versus stranger conditions (see Table 3 and Figure 2).

**TABLE 3:** Differences in child performance between parent present versus stranger present conditions.

	Reaction times		False alarm rate		Accuracy (hits)	
Main models						
	Estimate (95% CI)	*p* (adj. *p*)	Estimate (95% CI)	*p* (adj. *p*)	Estimate (95% CI)	*p* (adj. *p*)
Model 1	−1.05 (−3.93, 2.09)	0.501 (0.565)	−0.01 (−0.02, 0.00)	0.159 (0.477)	0.00 (0.00, 0.01)	0.565 (0.565)
Model 2	−1.05 (−3.93, 2.08)	0.501 (0.568)	−0.01 (−0.02, 0.00)	0.163 (0.489)	0.00 (0.00, 0.01)	0.568 (0.568)
Sensitivity analyses for puberty						
Model 2B	−1.10 (−5.03, 2.89)	0.608 (0.608)	−0.01 (−0.02, 0.01)	0.420 (0.608)	0.00 (0.00, 0.01)	0.589 (0.608)
Model 2C	−0.82 (−4.94, 3.56)	0.711 (0.711)	−0.01 (−0.02, 0.01)	0.351 (0.711)	0.00 (0.00, 0.01)	0.528 (0.711)

*Note*: Model 1 = controlled for emotion and block types effects, *N* = 501. Model 2 = controlled additionally for child biological sex assigned at birth and child age at task, *N* = 501. Model 2B = additionally controlled for pubertal stage, *N* = 268. Model 2C = children having entered puberty were removed, *N* = 240. All the models are performed excluding the go‐emotions in which the subject's accuracy was below 70%. The estimates provided are bootstrapped with 1000 bootstrap samples.

### 3.3 Sensitivity Analyses

In a smaller subsample, when controlling for the effects of puberty or excluding children who had entered puberty from the analyses (see Table 3, Models 2B and 2C; and Figure 2), we found no significant differences in child performance between the parent‐present and stranger‐present conditions. Similarly, in analyses stratified by sex, no significant differences in child performance between conditions were observed for either biological sex, whether considering the full sample or the sample controlling for pubertal stage or excluding children who had entered puberty.

In the main analysis, we excluded trials with low go‐trial accuracy (a criterion not specified in the pre‐registration, but consistent with general practices for this task). However, when performing analysis including all trials, there was tentative evidence for a difference in false alarm rates between the parent and stranger conditions. Specifically, children made fewer mistakes in the presence of a parent. The effect size of this finding resembled that of the main models, although the finding did not survive correction for multiple comparisons (estimate = −0.01 [−0.02, 0.00], *p* = 0.033, adjusted *p* = 0.10)

### 3.4 Modulation by Early Life Environmental Quality: Caregiving Quality and Maternal Psychological Distress During Childhood

There were no moderation effects of maternal caregiving quality, as measured by EA throughout early childhood, on child performance. These findings remained consistent when controlling for pubertal stage or excluding children who had entered puberty from the sample. Similarly, maternal psychological distress across childhood did not influence child performance in the full sample or in the subsample with available pubertal stage data, regardless of whether pubertal stage was controlled for or children who had entered puberty were excluded.

### 3.5 Exploratory Analyses: Paternal Psychological Distress During Childhood

Child performance was not significantly influenced by paternal distress across childhood when considering the full sample. In a subsample controlling for child pubertal stage, there was tentative evidence that paternal distress moderated differences in child false alarm rates (estimate = 0.02 [0.00, 0.03], p = 0.070). Although this effect strengthened when all trials were included regardless of go‐trial accuracy, it did not survive correction for multiple comparisons (estimate = 0.02 [0.00, 0.04], p = 0.023, adjusted p = 0.069), and the simple slopes for the conditions were not significant (see the Supporting Information).

## 4 Discussion

In this pre‐registered study, we utilized a large sample of families from backgrounds with relatively low socioeconomic and/or psychopathology risk (as indicated by high parental education, generally low parental distress, and a low proportion of mothers exhibiting low‐quality caregiving) to test whether parental presence is associated with 9.5‐year‐old children's performance on an emotional go/no‐go task, which is designed to assess emotion differentiation and regulation. Additionally, we examined the role of puberty for parental presence in influencing child performance, and whether long‐term environmental quality during childhood, measured as observed maternal caregiving quality assessed using EA Scales, and long‐term maternal (and exploratorily, paternal) psychological distress moderated children's task performance in the parental versus stranger conditions. At the specific age of 9.5 years, we found inconsistent evidence for a *parental buffering effect* (Gee et al. 2014; Hostinar et al. 2015) on child performance regardless of the child's pubertal status. Sensitivity analyses, including all trials regardless of go‐trial accuracy, suggested some improvement in child false alarm rates in the presence of a parent, with effect sizes similar across all models. This is consistent with our pre‐registered hypotheses; nevertheless, the effect size for this finding was modest and did not reach significance in the main models. We also found minimal support for the idea that normative variation in early life environmental quality modulates children's reliance on parental presence. By leveraging a large sample and longitudinal design, these findings contribute to a more nuanced understanding of the parental role in children's emotion regulation just before pubertal transition and provide targeted directions for future research. However, given the limited variation in pubertal status among participants in our study, the results related to puberty should be considered preliminary.

Although some inconsistencies in the findings emerged depending on the sample selected and the data‐management criteria applied, it appears that 9.5‐year‐old children from these low‐risk backgrounds, regardless of their pubertal status, are not strongly dependent on parental buffering for emotion‐related task performance, especially with regard to their performance accuracy or reaction times. Interestingly, when all trials were included regardless of the go‐accuracy, children made fewer mistakes in the presence of the parent compared to a stranger, with effect sizes similar across all models. This finding is consistent with our pre‐registered hypothesis and prior literature, suggesting that *if* parental presence plays a role at this age, it tends to be beneficial for child performance.

This set of findings is a notable contribution, as few prior studies have focused on the role of parental presence on behavioral indicators of self‐regulation in a large general population sample. Many existing studies have relied on smaller samples, often due to the inclusion of parallel neuroimaging components, or have focused on children from high‐to extreme‐risk backgrounds—contexts known to significantly influence the development of self‐regulation (IJzendoorn et al. 2020; Johnson et al. 2021; Miu et al. 2022). Moreover, prior research has frequently included broad age ranges, limiting the ability to detect age‐specific patterns or subtle developmental shifts in the role of parental presence. However, although the narrow age range of the sample is a strength, it also limits the generalizability of our findings to broader age groups, including younger or older individuals or samples with a larger range of pubertal statuses. Therefore, it is critically important for future research to continue examining the development of parental buffering across early and middle childhood.

Interestingly, when analyzing a subsample with available information on pubertal stage, we were unable to detect differences between the parental and stranger conditions regardless of pubertal status. While this analysis—conducted in a smaller subsample that required excluding some positive pubertal assessments that occurred after the behavioral task—warrants cautious interpretation and remains preliminary, it further suggests that parental presence may generally play a lesser role in children aged 9–10 years. This finding contrasts, at least in part, with prior studies indicating that puberty is a key mechanism modulating the parental role in regulating children's performance and stress physiology (Doom et al. 2015; Gee et al. 2014) and implies that other developmental mechanisms may have a greater influence on parental buffering during the period just before the transition to puberty.

These results underscore the need for sufficiently large samples with repeated assessments from early childhood through adolescence to better understand the nuanced mechanisms shaping the developmental trajectory of parental buffering. The tentative finding that children made fewer errors in the presence of a parent also suggests that children with particularly poor or variable performance may represent a subgroup worth exploring in future studies of parental buffering during emotional tasks. However, we could not specify this criterion at the pre‐registration stage, which represents a limitation of the present study. Future research using go/no‐go tasks should clearly define data‐cleaning criteria at pre‐registration to ensure the integrity and transparency of the analyses.

Consistent with previous studies suggesting that early life adversity may accelerate development by modulating both the timing of puberty (Hamlat et al. 2023; MacSweeney et al. 2025; Pham et al. 2022; Thijssen et al. 2020) and the child's reliance on caregivers for emotion regulation and related brain networks (Doom et al. 2015; Gee et al. 2013; Thijssen et al. 2017), we explored whether normal variation in environmental quality across childhood influences parental buffering of child task performance. However, we found very little support for this hypothesis, as neither the pre‐registered analysis of maternal caregiving and mothers’ long‐term distress, nor the exploratory analysis of fathers’ long‐term distress indicated that these exposures consistently moderated child performance across conditions. This may reflect the nature of our low‐risk sample, where environmental quality may not be “adverse enough” to modify children's detachment from parental buffering, or potentially affect the underlying timing of puberty. Furthermore, because the puberty data were available only for a subsample of children, we were unable to fully explore the role of these environmental factors in relation to pubertal timing within the scope of this study. Future studies using the larger source cohort, including more comprehensive puberty data, will be better positioned to address these questions in detail. We also lacked data on paternal caregiving quality and, therefore, could not examine the potential role of the father–child relationship quality, which remains an important avenue for future research to explore.

There are several notable strengths in the study, including a large sample size, a more narrowly defined age range compared to previous studies, allowing for more specific conclusions, and longitudinal, repeated measures of caregiving and parental psychological distress. One limitation of the study is the absence of pubertal data for half of the sample, due to synchronization challenges in visit timing, such as those caused by the COVID‐19 pandemic affecting data collection. Furthermore, the sample had a narrow range of pubertal statuses, limiting the ability to fully examine pubertal effects. This limitation precludes conclusions about how much of the lack of parental presence effects can be attributed to the many children who had already entered puberty. Future research using the same cohort will have increased statistical power to examine how early life factors influence pubertal timing, which was not within the scope of the current study. Nevertheless, repeated assessments of the emotional go/no go task with parental presence would help disentangle the complex interactions between early life factors such as parental distress, early puberty development, and emotion regulation. Another limitation is the timing of the emotional go/no‐go task, which was administered toward the end of a relatively long developmental visit. This may have influenced some children's performance, particularly when compared to other experimental studies where children complete only one task. However, because conditions were counterbalanced between parent and stranger sessions, and children were given adequate breaks throughout the visit, this potential confound is unlikely to have significantly affected the observed session effects. Finally, we did not include concurrent measures of attachment or parent–child interaction in the current manuscript, as these data were not yet fully available. Examining these measures would have also been beyond the scope of the current study, which primarily focused on early life environmental quality. Future analyses, will incorporate both concurrent measures of the environment and attachment, as well as neuroimaging data from a subsample of participants in the current dataset.

In conclusion, our findings suggest that there is no consistent evidence for parental buffering of behavioral task performance in 9.5‐year‐old children from backgrounds with low socioeconomic and psychopathology risk, regardless of pubertal status. However, sensitivity analyses—showing effect sizes similar to those in the main models—indicated that children made fewer errors when a parent, rather than a stranger, was present. This finding aligns with previous research and our pre‐registered hypothesis. Our findings have important implications for future research, highlighting the need to examine buffering effects across childhood in greater detail, with careful consideration of the various developmental mechanisms, performance variability profiles, and underlying neural processes involved. Such an approach will help clarify the mechanisms driving shifts in emotional regulation throughout childhood. Notably, the age of 9 years and above may be too high for observing consistent parental buffering effects, yet too low to fully capture pubertal influences. Additionally, we found little evidence that typical variations in caregiving quality or parental distress influence children's reliance on their caregivers. This finding is somewhat reassuring, considering the extensive literature on the impact of early environmental factors—including parental mental health and caregiving quality—on the development of emotion regulation and parent–child relationship dynamics.

## Author Contributions

**Laura Perasto**: formal analysis, visualization, writing – review and editing. **Hilyatushalihah Kholis Audah**: investigation, project administration, writing – review and editing. **Hasse Karlsson**: funding acquisition, supervision, writing – review and editing. **Max Karukivi**: writing – review and editing, conceptualization. **Tuomo–Artturi Autere**: data curation, writing – review and editing. **Minna Lukkarinen**: funding acquisition, supervision, writing – review and editing, methodology. **Nim Tottenham**: conceptualization, methodology, supervision, writing – review and editing. **Saara Nolvi**: conceptualization, funding acquisition, methodology, project administration, supervision, writing – original draft, writing – review and editing. **Venla Huovinen**: data curation, investigation, project administration, writing – review and editing. **Pauliina Juntunen**: data curation, project administration, writing – review and editing, investigation. **Aino Luotola**: investigation, project administration, writing – review and editing. **Riikka Korja**: conceptualization, methodology, supervision, funding acquisition, writing – review and editing. **Linnea Karlsson**: funding acquisition, supervision, writing – review and editing. **Anna–Katariina Aatsinki**: funding acquisition, methodology, project administration, supervision, writing – review and editing.

## Funding

This research was funded by Research Council of Finland (346121, 347640, 253270, 134950, 264363, 314390, 332444), Strategic Research Council (SRC) established within the Research Council of Finland (35264, 362655), Signe and Ane Gyllenberg Foundation, State Grants for Clinical Research (VTR), Emil Aaltonen Foundation, Finnish Cultural Foundation, Juho Vainio Foundation, Jane and Aatos Erkko Foundation, Brain and Behavior Research Foundation (1956) and Stiftelsen Eschnerska Frilasarettet.

## Conflicts of Interest

The authors declare no conflicts of interest.

## Supporting information




**Supporting File1**:desc70206‐sup‐0001‐SuppMat.docx.

## Data Availability

Current EU and national legislation on personal data protection of sensitive data and the informed consents given by the study subjects do not permit open data sharing. Investigators interested in research collaboration and obtaining access to the data are encouraged to contact the FinnBrain board (finnbrain‐board@utu.fi). Contact information of the board members or FinnBrain's Principal Investigators is listed on the project website: https://sites.utu.fi/finnbrain/en/contact/.
