# Supplementary material for: Does Parental Presence Influence Child Performance on an Emotional Go/No‐Go Task at Age 9.5? Exploring the Role of Puberty and Early Environmental Quality
Source: Dev Sci. 2026 Apr 26;29:e70206. doi: 10.1111/desc.70206 (PMC13111780; doi:10.1111/desc.70206)
Supplement: Supplementary file 1 — Supporting File1:desc70206‐sup‐0001‐SuppMat.docx. [file DESC-29-e70206-s001.docx]

**Supplement**

1. **The Maternal EA Direct Scores at Different Time Points**

**Table S1.** The maternal EA direct scores across subscales and time points (theoretical range 1-7, with values below 3.0 reflecting highly problematic interaction)

|  | Range | Mean | Std. Deviation |
| --- | --- | --- | --- |
| 5 years |  |  |  |
| Sensitivity | 2.5-7.0 | 5.34 | 0.93 |
| Structuring | 2.5-7.0 | 4.94 | 0.92 |
| Non-intrusiveness | 3.0-7.0 | 5.92 | 0.81 |
| Non-hostility | 4.0-7.0 | 6.26 | 0.75 |
| 30 months |  |  |  |
| Sensitivity | 2.5-7.0 | 5.21 | 1.09 |
| Structuring | 2.5-7.0 | 5.36 | 1.14 |
| Non-intrusiveness | 3.0-7.0 | 5.83 | 1.21 |
| Non-hostility | 3.0-7.0 | 6.39 | 0.83 |
| 8 months |  |  |  |
| Sensitivity | 3.0-7.0 | 5.25 | 1.31 |
| Structuring | 2.0-7.0 | 5.03 | 1.48 |
| Non-intrusiveness | 2.0-7.0 | 5.63 | 1.37 |
| Non-hostility | 3.0-7.0 | 6.16 | 1.05 |

1. **The Correlations between the Main Study Variables across Parental and Stranger Sessions and Across the Three Emotional Stimuli**

**Table S2.** The associations between the main study variables across sessions and different emotions

| **With the Stranger** | **1** | **2** | **3** | **4** |
| --- | --- | --- | --- | --- |
| 1 Reaction time | 1 |  |  |  |
| 2 False alarm rate | -0.360*** | 1 |  |  |
| 3 Accuracy (Hits) | -0.077*** | 0.077*** | 1 |  |
| 4 Age of the child | -0.060** | -0.007 | -0.024 | 1 |
| 5 Sex of the child^a^ | 0.204*** | -0.199*** | 0.072*** | -0.051** |
| **With the Parent** |  |  |  |  |
| 1 Reaction time | 1 |  |  |  |
| 2 False alarm rate | -0.319*** | 1 |  |  |
| 3 Accuracy (Hits) | -0.063** | 0.061** | 1 |  |
| 4 Age of the child | -0.030 | -0.001 | 0.019 | 1 |
| 5 Sex of the child^a^ | 0.181*** | -0.181*** | 0.064** | -0.044* |
| **Emotion = angry** |  |  |  |  |
| 1 Reaction time | 1 |  |  |  |
| 2 False alarm rate | -0.372*** | 1 |  |  |
| 3 Accuracy (Hits) | -0.120*** | 0.285*** | 1 |  |
| 4 Age of the child | -0.042 | 0.023 | 0.016 | 1 |
| 5 Sex of the child^a^ | 0.186*** | -0.194*** | 0.065** | -0.043 |
| **Emotion: Happy** |  |  |  |  |
| 1 Reaction time | 1 |  |  |  |
| 2 False alarm rate | -0.322*** | 1 |  |  |
| 3 Accuracy (Hits) | -0.019 | 0.045 | 1 |  |
| 4 Age of the child | -0.023 | 0.002 | -0.007 | 1 |
| 5 Sex of the child^a^ | 0.188*** | -0.196*** | 0.105*** | -0.042 |
| **Emotion: Fear** |  |  |  |  |
| 1 Reaction time | 1 |  |  |  |
| 2 False alarm rate | -0.365*** | 1 |  |  |
| 3 Accuracy (Hits) | -0.055* | -0.026 | 1 |  |
| 4 Age of the child | -0.074** | -0.036 | -0.016 | 1 |
| 5 Sex of the child^a^ | 0.205*** | -0.191*** | 0.044 | -0.057* |

1. **Difference between Parent vs. Stranger Conditions: General Linear Models (as Outlined in the Pre-Registration)**

The main results using more traditional General Linear Models are presented in Table S3. Results closely resemble the findings obtained using linear mixed models.

**Table S3.** The results of general linear models for difference between parent vs. stranger conditions using similar models as in the main manuscript

|  | Reaction times | | False alarm rate | | Accuracy (Hits) | |
| --- | --- | --- | --- | --- | --- | --- |
| Main models | | |  | |  | |
|  | Estimate (95% CI) | P (adj. p.) | Estimate (95% CI) | P (adj. p.) | Estimate (95% CI) | P (adj. p.) |
| Model 1 | -1.05 (-4.76, 2.71) | .575 (.592) | -0.01 (-0.02, 0.00) | .210 (.592) | 0.00 (0.00, 0.01) | .592 (.592) |
| Model 2 | -1.04 (-4.75, 2.71) | .577 (.591) | -0.01 (-0.02, 0.00) | .210 (.591) | 0.00 (0.00, 0.01) | .591 (.591) |
| Sensitivity analyses for puberty | | |  |  |  |  |
| Model 2B | -1.17 (-6.27, 4.31) | .670 (.670) | 0.00 (-0.02, 0.01) | .598 (.670) | 0.01 (0.00, 0.01) | .337 (.670) |
| Model 2C | -0.61 (-6.01, 4.61) | .787 (.787) | 0.00 (-0.02, 0.01) | .581 (.787) | 0.00 (0.00, 0.01) | .274 (.787) |
| Model 1 = controlled for emotion and block types effects, N = 501  Model 2 = controlled additionally for child biological sex assigned at birth and child age at task, N = 501  Model 2B = additionally controlled for pubertal stage, N = 268  Model 2C = children having entered puberty were removed, N = 240  All the models are performed excluding the go-emotions in which the subject’s accuracy was below 70%. The estimates provided are bootstrapped with 1000 bootstrap samples. Multiple comparison correction (three outcomes) was performed for each model level using FDR correction. | | | | | | |

1. **Associations between Background Factors, Early Life Environmental Quality Variables, and Child Emotional Go/No-Go Performance**

Early Life Environmental Quality. The selected maternal background factors (maternal education, economic satisfaction and age) were modestly associated with maternal EA and maternal distress as well as some child outcomes (Table S4) so all were controlled for in the models. Similarly, paternal background factors were related to paternal distress as well as some child outcomes and thus were included in all the subsequent models (Table S5).

Table S4. Spearman correlations coefficients between maternal background variables and child performance outcomes

|  | 1. | 2. | 3. | 4. | 5. | 6. | 7. |
| --- | --- | --- | --- | --- | --- | --- | --- |
| 1. Education | 1 |  |  |  |  |  |  |
| 2. Fin. satisfaction | 0.22*** | 1 |  |  |  |  |  |
| 3. Age | 0.20*** | -0.03*** | 1 |  |  |  |  |
| 4. Distress | -0.08*** | -0.30*** | -0.07** | 1 |  |  |  |
| 5. Maternal EA | 0.11*** | 0.02*** | -0.08*** | 0.03 | 1 |  |  |
| 6. Reaction time | 0.01*** | 0.09*** | 0.03* | -0.09*** | 0.05** | 1 |  |
| 7. False alarm rate | -0.08*** | -0.10** | 0.00 | 0.06* | -0.09*** | -0.32*** | 1 |
| 8. Accuracy | 0.03 | -0.04 | 0.06** | -0.01 | 0.02 | -0.09*** | 0.09*** |

Note: ***p<.001, **p<.01, *p<.05.

Table S5. Spearman correlation coefficients between paternal background variables and child performance outcomes

|  | 1 | 2 | 3 | 4 | 5 | 6 |
| --- | --- | --- | --- | --- | --- | --- |
| 1 Education | 1 |  |  |  |  |  |
| 2 Fin. satisfaction | 0.16*** | 1 |  |  |  |  |
| 3 Age | 0.03*** | 0.01 | 1 |  |  |  |
| 4 Distress | -0.05** | -0.14*** | -0.03 | 1 |  |  |
| 5 Reaction time | 0.08*** | 0.00 | 0.04* | 0.01* | 1 |  |
| 6 False alarm rate | -0.06*** | 0.00 | -0.07*** | 0.00 | -0.33*** | 1 |
| 7 Accuracy | -0.02 | -0.03 | -0.02 | 0.03 | -0.10*** | 0.11*** |

Note: ***p<.001, **p<.01, *p<.05.

1. **Early Life Environmental Quality Moderation Figures and Simple Slope Analysis**

**Paternal Psychological Distress.** Paternal psychological distress modulation for session difference in child false alarm rates across different samples is shown in Figure S1 (a = whole data with data accuracy criterion applied; b = pubertal stage controlled for; c = whole data with accuracy criterion for go trials not applied; d = children in puberty excluded, and accuracy criterion not applied). Based on figure and interaction analysis, long-term paternal distress was associated with fewer errors when alone compared to parent present. However, simple slope analyses were not significant, indicating no significant association between paternal distress and false alarm rates in separate conditions (parent vs. stranger).


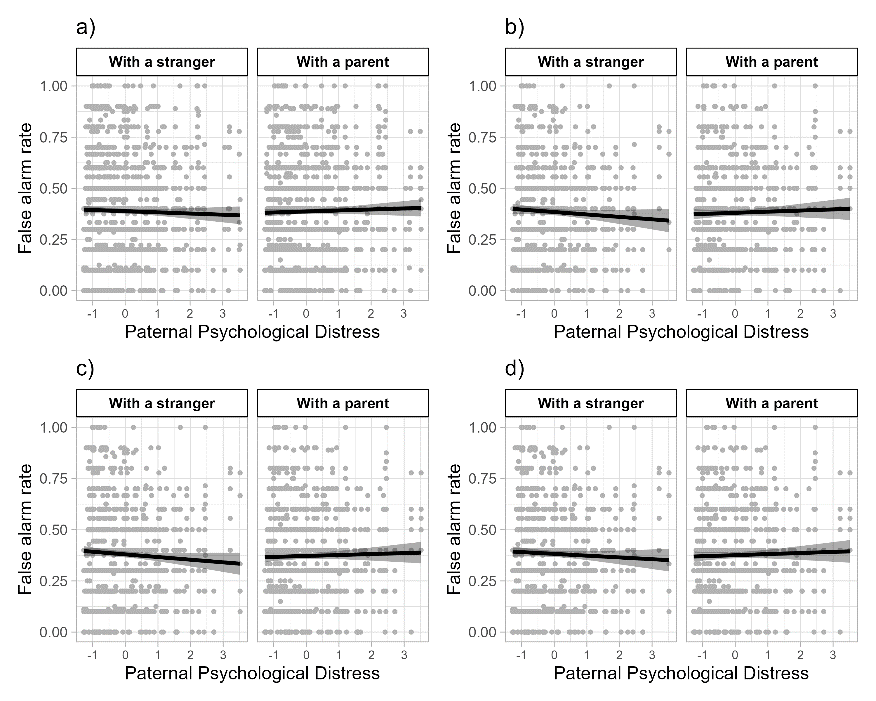


**Figure S1.** The modulation of child false alarm rates by paternal long-term distress across childhood; a = whole data with data >70% go accuracy criterion applied; b = pubertal stage controlled for; c = whole data with all go trials included in the analysis; d = children in puberty excluded, and all go trials included in the analysis

1. **Early Life Environmental Quality Moderation: General Linear Models (as Outlined in the Pre-Registration)**

**Maternal caregiving quality and psychological distress.** Consistent with linear mixed modelling approach, there was no modulation of parent vs. stranger condition effect on child performance by maternal caregiving as measured by EA or maternal psychological distress throughout childhood.

**Paternal psychological distress.** Consistent with linear mixed models, and including all trials regardless of the go trial accuracy, there were no modulation effects by paternal distress when the full dataset was considered. In a subsample controlling for pubertal stage, the tentative moderation effect by paternal distress also diminished when using GLM (Estimate = 0.02[0.00,0.04], p = .092, adjusted p = .276).
